# Sing More

**Published:** 1873-03

**Authors:** 


### SING MORE.

Cultivate singing in the family. Begin when the child is not yet three years old. The songs and hymns your mother sang, bring them all back to your memory, and teach them to your little ones; the hymn and the ballad; funny and devotional; mix them altogether, to meet the similar moods, as in after life they come over us so mysteriously sometimes. Many a time and oft, in Wall street and Broadway, in the very whirl of business; in the sunshine and gayety of Fifth Avenue, and amid the splendor of the drives in the Central Park, some little thing wakes up the memories of early youth—the old mill; the cool spring; the shady tree by the little school-house—and the next instant we almost see again the ruddy cheek, the smiling faces, and the merry eyes of schoolmates, some grey-headed now, most "lie moldering in the grave." And anon, "the song my mother sang" springs unbidden to the lips, and soothes and sweetens all these memories.

At other times, amid the crushing mishaps of business, a merry ditty of the olden time pops up its little head, breaks in upon the ugly train of thought, throws the mind into another channel; light breaks in from behind the cloud in the sky, and a new courage is given to us. The honest man goes singing to his work; and when the day's labor is done, his tools laid aside, and he is on his way home, where wife and child, and tidy table and cheery fireside await him, he cannot help but whistle or sing.

The burglar never sings. Moody silence, not the merry song, weighs down the dishonest tradesman, the perfidious clerk, the unfaithful servant, the perjured partner.

The very sound of music brings back the memories of childhood, its joyous innocence, and stays the head and hand from crime and wrong doing, when the poor tempted one was almost ready to fall.

Music promotes purity and elevation and culture and joyousness and mirth; and who does not know that mirth promotes health? Music deepens the breathing; it enlivens the circulation; dispels the congestion of the body and the brain, and notably strengthens the lungs.

Landing among the ridiculous has a certain degree of comfort with it, although one has just come from heights sublime; it breaks the fall!! The sudden transition from what is serious to something which convulses with laughter, is many a time of incalculable advantage. Hence, besides psalms and hymns, children should be taught scraps of poetry, in snatches or verses, the more utterly absurd or whimsical or nonsensical, the better. It is not the sense we want, but the diversion. Most readers, before now perhaps, have been in companies larger or smaller, under very depressing circumstances; a cloud on every brow; faces lugubrious to the last degree, or as long as fence rails; when all at once some merry fellow strikes up a note, or repeats a joke, or rehearses a piece of poetry, and in an instant the whole company is electrified; that brings out another joke or jest. The more unexpected, the greater the fun. For example: It is a pitiless falling rain, just at dark, a November evening, within a mile or two of home; a whole car-load of passengers are waiting for a repair of the track; have waited an hour; the information comes in a cheery tone from some wag, " Ladies and gentleman, the superintendent desires me to inform you, that the obstruction will be removed most certainly—sooner or, later, especially the latter; let us 'sing,'" and away he goes, with an inimitable squeak:

> O once I loved a purty girl,
> Her name it was Marier;
> But Polly dear, my love to you,
> Is forty-five times higher!
> Riddle ding a day,
> Ding, a dy-ding adoho;
> Riddle, ding a day ding
> A die ding a doho.

Music is refining; it is a safeguard against vice and crime and every wrong doing; it promotes sociability as well as devotion; it brings people together. It helps to while away the time wonderfully in travel, especially in steamboats, and at night in country places. In traveling through glorious New England, it will often be noticed, that one or two persons in a company will begin to sing, others gather round, gain courage to join in, and before one knows it a chorus will strike up through the boat and make the whole cabin reverberate above the noise of machinery, the dashing of the waters, and the fury of the storm. There was a little boy on board going up the Saguenay, last summer, with a crowd of people; the night was dark, and everybody seemed to want something to turn up. So we turned up the little boy, not more than a dozen years old; by standing by him and giving him countenance, being old friends, he sat down to the piano and played away bravely.

After that followed a jovial young lady from Boston:

Oh did you know the muffin man,
The muffin man, the muffin man,
Oh did you know the muffin man,
That lived in Drury Lane?

Oh, yes I know the muffin man,
The muffin man, the muffin man,
Oh yes I know the muffin man,
That lived in Drury Lane.

Then it most provokingly stopped, you naturally expected to hear something about him, but didn't, yet, by dint of repetition, in a few moments there was a general joining in with the chorus.

Then another began the long story about the straits of Belle Isle, running thus:

At the straits of Belle Isle,
I was there all the while,
I was there all the while,
At the straits of Belle Isle.
All the while I was there,
At the straits of Belle Isle,
I was there all the while,
At the straits of Belle Isle.

After repeating the above a long time in a sweet voice we became more and more anxious to know something further about the straits of Belle Isle; the information was given, "That's all."

The absence of sense in all these provoked mirth, as well as the very absurdity of the idea; having the effect further to bring the passengers together and make them acquainted.

This, by the way, is one of the elements which makes the

New England watering places so much more thoroughly enjoyable than others ; there seems to be a feeling furthermore, that something is to be aimed at better than the display of dresses and diamonds ; there is more that is homelike and enjoyable; enough of politeness to maintain a proper degree of respectful consideration, but not so much of form and ceremony as to freeze everything into icicles. Hence, the crowds which throng to New England watering places every summer up to the last day, to be closed with regret, and to be reverted to for long years thereafter with pleasant memories.

One of the most delightful of pictures is that of a family of sons and daughters and parents, with a friend or two, gathering around the homestead fireplace, of a Sunday evening or winter's night, and singing in unison one of the "songs of Zion," carrying all the parts. If this thing is done as a habit, not on Sunday nights only, but often through the week, especially when company comes—the young folks of neighbors—there will be an inexpressible delightfulness in it, and that family will grow up in a harmony and unity and lovingness, which is altogether impossible to those who never have such representations of heaven around their firesides, but prefer spending their evenings in games of cards, in novel reading in their solitary chambers, and associations with others than those of the home circle. As a source of safety, of happiness, of purity, and as a means of promoting family unity and affection, and inviting children away from the street and evil associations, let parents sing more, and make it a matter of duty and conscience and love, to teach all their children to sing, and do it early, beginning at the very first year or two of life with the songs of the church.

